# Microfluidic approaches for the fabrication of gradient crosslinked networks based on poly(ethylene glycol) and hyperbranched polymers for manipulation of cell interactions

**DOI:** 10.1002/jbm.a.32974

**Published:** 2011-01

**Authors:** S Pedron, C Peinado, P Bosch, J A Benton, K S Anseth

**Affiliations:** 1 Instituto de Ciencia y Tecnología de Polímeros, CSIC, Juan de la Cierva 328006 Madrid, Spain; 2 Department of Chemical and Biological Engineering, University of ColoradoECCH 111, UCB 424, Boulder, Colorado 80309-0424; 3 The Howard Hughes Institute, University of ColoradoECCH 111, UCB 424, Boulder, Colorado 80309-0424

**Keywords:** hyperbranched polymers, cell morphology, valvular interstitial cells, elasticity, surface topography, gradient substrate

## Abstract

High-throughput methods allow rapid examination of parameter space to characterize materials and develop new polymeric formulations for biomaterials applications. One limitation is the difficulty of preparing libraries and performing high-throughput screening with conventional instrumentation and sample preparation. Here, we describe the fabrication of substrate materials with controlled gradients in composition by a rapid method of micromixing followed by a photopolymerization reaction. Specifically, poly(ethylene glycol) dimethacrylate was copolymerized with a hyperbranched multimethacrylate (P1000MA or H30MA) in a gradient manner. The extent of methacrylate conversion and the final network composition were determined by near-infrared spectroscopy, and mechanical properties were measured by nanoindentation. A relationship was observed between the elastic modulus and network crosslinking density. Roughness and hydrophilicity were increased on surfaces with a higher concentration of P1000MA. These results likely relate to a phase segregation process of the hyperbranched macromer that occurs during the photopolymerization reaction. On the other hand, the decrease in the final conversion in H30MA polymerization reactions was attributed to the lower termination rate as a consequence of the softening of the network. Valvular interstitial cell attachment was evaluated on these gradient substrates as a demonstration of studying cell morphology as a function of the local substrate properties. Data revealed that the presence of P1000MA affects cell–material interaction with a higher number of adhered cells and more cell spreading on gradient regions with a higher content of the multifunctional crosslinker.

## INTRODUCTION

The interaction of cells with materials is an extremely complicated subject, but one that is of high importance in both cell biology and biomaterial design.^1^ The studies of cellular responses to substrates suggest that cells are sensitive to the chemical functionality, topography and underlying mechanics.^2–4^ One important problem in studies comparing different types of polymers is that the compositions can be heterogeneous both chemically and physically (e.g., different surface chemistry, charge, roughness, rigidity, and crystallinity), which may result in considerable variation in experimental results. Another methodological problem is that the evaluation of cell function on polymer surfaces is often tedious, because of the large number of samples and the preparation that is required to study the range of desired parameters of interest. Thus, there is growing interest in techniques to fabricate substrates with gradient properties that can be used to explore cell–material interactions in a continuum fashion.^5^ A number of research groups have focused on the preparation of substrates with a gradually varying chemical composition along one dimension,^6–8^ and these “gradient surfaces” are providing facile approaches to screen and identify the effects of specific materials properties on basic cell functions.

Numerous studies have described the influence of substrate chemical composition on the behavior of neurons^9^, ^10^ (e.g., migration or axon orientation) or myoblasts^11^ but these approaches often rely on complex and experimentally intensive techniques for gradient production. Several techniques have been developed to generate biomaterials with gradients in either one or two directions.^12–14^ An example of the fabrication of one-dimensional gradient materials was demonstrated by Simon et al.^15^ who manufactured strip-shaped gradients in polymer blend composition of poly(l-lactic acid) and poly(D,l-lactic acid). No difference in cell adhesion was observed, although there were distinct differences in the rate of cell proliferation across the material depending on surface roughness. Gradients of polymer crystallinity created by annealing poly(lactic acid) on a temperature gradient have demonstrated that cells are exquisitely sensitive to variations in nanometer-scale topography.^16^ The effect of surface energy and hydrophilicity on fibronectin-mediated cell adhesion, spreading and proliferation have been studied by the fabrication of surface energy gradients by exposure of a self-assembled monolayer to UV light in a graded fashion.^17^ More recently, the fabrication of hydrogels with variations in elasticity has shown that decreasing the modulus of the substrate reverses valvular interstitial cells (VICs) activation to myofibroblasts.^18^

Microfabricated fluidic devices can also be used to generate concentration gradients.^19^ A microfluidic gradient generator consisting of multiple generation branches in a poly(dimethylsiloxane) network was fabricated by rapid prototyping and soft lithography. Wang and coworkers^20^ were among the first groups to create such substrates by interdiffusing mixtures of acrylamide and bis-acrylamide of different compositions. Wong and coworkers^21^, ^22^ extended this method by using photopolymerization in combination with either micropatterning or microfluidics to fabricate substrates with microscale control over the mechanical properties by exposing acrylamide and bis-acrylamide solutions to UV light across masks having position dependent shading. By both methods, a cell response to substrate elasticity was observed, specifically a preferential migration of cells onto stiffer regions of substrates. The microfluidic systems are also useful methods to study cell responses to gradients of soluble factors; cells can be cultured in channel systems and the compound transiently infused to the chip to generate concentration gradients.^23^ Conversion gradient substrates have been used to evaluate the effects of methacrylate conversion on cell response, with both local leachables and the under-cured polymer network potentially affecting cellular response.^24^

Two studies have used high-throughput analyses to determine the reaction kinetics^25^ and reactivity ratios of free radical co-polymerizations.^26^ By using this methodology, linear gradients in crosslinking density were developed by addition of increasing concentrations of a multifunctional crosslinker. Hyperbranched polymers provide unique properties, such as good solubility, low viscosity and high functionality, due to their 3D architecture; thus, the materials formed thereof achieve better biological response with tailored mechanical properties and integrin-mediated cell adhesion. Here, we investigate the mechanical properties, hydrophilicity and surface roughness of gradient materials formed from the copolymerization of two multimethacrylate macromers, and study the influence of these properties on the attachment of VICs.

## MATERIALS AND METHODS

### Materials

Poly(ethylene glycol) dimethacrylate (PEGDM, *M*_n_ 550 Da) was purchased from Aldrich. Hyperbranched polymer Hybrane® P1000 with di-2-propanolamine end groups and a building group of phthalic anhydride was kindly provided by DSM, The Netherlands.^27^ Boltorn® H30 has an ethoxylated pentaerythritol moiety as a central core and 2,2-bis(methylol)propionic acid (bis-MPA) as dendritic units and was generously provided by Perstorp, Sweden.^28^ The photoinitiator bis-(2,4,6-trimethylbenzoyl)-phenylphosphine oxide (Irgacure 819®) was provided by Ciba Specialty Chemicals, Basel, Switzerland, and used as received. Modification of the hyperbranched polymer was carried out by esterification of hydroxyl groups (an average of 7 for P1000 and 32 for H30) with methacryloyl chloride (P1000MA and H30MA). The hyperbranched polymer (1 mmol based on hydroxyl end groups) and *N*,*N*-dimethylaminopyridine (0.05 mmol) were dissolved under nitrogen in dry dichloromethane and triethylamine (1.5 mmol) in a reaction vessel. Methacryloyl chloride (1.5 mmol), freshly distilled, diluted with dichloromethane was slowly added to the mixture at 0°C, and the solution was left stirring at room temperature overnight. The solution was extracted twice with aqueous HCl (2%) and NaOH (2%) solutions, then dried over anhydrous MgSO_4_, filtered, and finally evaporated, yielding a slightly yellow viscous product. The methacrylation efficiency was confirmed by ^1^H NMR. P1000MA and H30MA had an average of 5 and 16 methacrylate reactive groups, respectively.

### Preparation of gradient samples

The experimental setup is described in detail elsewhere,^29^ but a brief overview is provided here. Composition gradients were produced using a microfluidic mixer, controlled by two syringe pumps (Harvard Apparatus Model #980499), to control the monomer flow rates and ultimately the comonomer composition. Gradient formation involves the injection of two monomer solutions into a unique gradient generator that consists of a network of microchannels that mix the injected solutions. Monomer solutions of 8 wt % hyperbranched macromer concentration (H30MA or P1000MA) in PEGDM and pure PEGDM monomer were used. After passing through the gradient maker, the monomer solutions enter a larger viewing channel (70 × 2 × 1 mm^3^), where a stable gradient was formed, and the solutions were photopolymerized to form a crosslinked network upon exposure to ultraviolet light exposure (Novacure, 100 W Hg, 5 mW/cm^2^). Each side of the sample was exposed for 10 min, and the final methacrylate conversion was measured with FT-NIR.

### FT-NIR analysis

The gradient samples were placed in a Fourier transform infrared (FTIR) microscope (Nicolet Continuμm®). FTIR spectra were measured at 4 cm^−1^ resolution with four spectrum scans per point using the IR microscope. Data acquisition was carried out by software Atlμs (Nicolet Instruments corp.). The positions of the edges of the sample and each point were known and sampled sequentially by the microscope, collecting data every 500 μm. The conversion was calculated as a function of position across the entire gradient using NIR wavelengths. Spectra were collected before and after curing, and the final conversions were calculated as the reduction in the vinyl peak at 6162 cm^−1^. The polymer composition was also determined as a function of the aromatic (4623 cm^−1^) and hydroxyl group (4872 cm^−1^) absorption for P1000MA and H30MA respectively, taken along the conversion gradient. Values were averaged at the same position for three different strip samples. The standard uncertainty associated with the NIR measurements is ∼3%.

### Surface characterization using contact angle and AFM

The contact angle as a function of position across the gradient surfaces was measured at 25°C by the “sessile drop” method using water as the probe fluid and a CAM200 KSV tensiometer. The standard uncertainty is represented by the standard deviation between three independent measurements every 10 mm. Tapping-mode atomic force microscopy (AFM) measurements were conducted in air with a Nanoscope IV system (Digital Instruments). The root-mean-square (rms) roughness was determined using standard Digital Instruments software, and average and standard deviations were calculated from multiple measurements every 10 mm along the gradient substrates.

### Nanoindentation

Nanoindentation measurements were performed using an MTS Nanoinstruments NanoXP instrument (Oak Ridge, TN) equipped with a 1-μm Berkovitch indenter. The continuous stiffness method, using 45 Hz, 5 nm dynamic oscillations, was used to determine the elastic modulus continuously throughout the loading portion of the experiment. With the area function of the indenter tip determined from calibration procedures in a known standard, Eqs. 1 and 2 were used to calculate the hardness and reduced elastic modulus (*E*_r_), respectively. P_max_ is the maximum load, S is the contact stiffness, A the contact area and β the geometry correction factor (1.034). The sample modulus (*E*) was then calculated with knowledge of the sample's Poisson's ratio (ν), indenter elastic modulus (*E*_i_), and indenter Poisson's ratio (ν_i_) [Eq. 2]: 
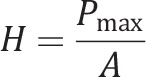
1

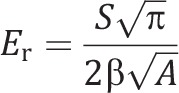
2

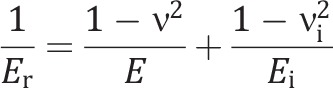
3

The reported values of the modulus are an average of the moduli obtained over a depth range from 1000 to 4000 nm. All indentation experiments were conducted using a strain rate of 0.05 s^−1^. The value of Poission's ratio was assumed to be 0.5 because of the elastomeric character of the network. Data is presented as the mean ± standard deviation of nine indentations.

### VIC culture

Aortic valve leaflets were surgically isolated from porcine hearts purchased from Quality Pork Processors (Austin, MN), and VICs were isolated by sequential collagenase digestion as previously described.^30^ VICs were cultured in growth media consisting of 15% fetal bovine serum (FBS), 2% penicillin/streptomycin, 0.4% fungizone, 0.2% gentamicin in Media 199 (Invitrogen Corp., Carlsbad CA) at 37°C in a 5% CO_2_ environment. VICs were cultured in 1% fetal bovine serum-supplemented medium to minimize cell proliferation and seeded at a concentration of 50,000 cells/cm^2^.

### Data analysis

Data is presented as mean ± standard deviation. At a minimum, three samples were averaged for each data point. Data was compared using a two-tailed, unpaired *t* test, and *p* values less than 0.05 were considered statistically significant.

## RESULTS AND DISCUSSION

**Figure 1:**
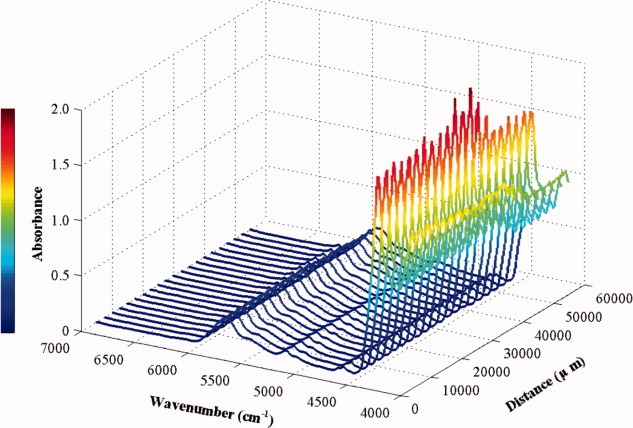
NIR spectra illustrating the PEGDM/P1000MA composition along the length of the gradient polymer substrate after UV exposure. The hyperbranched macromer (P1000MA) concentration increases with distance. For clarity, only every fifth spectrum is shown (2.5 mm increments). The peak at 6162 cm^−1^ is due to the methacrylate {C}H and 4623 cm^−1^ to the aromatic C}H absorbance.

Gradient materials were fabricated using a microfluidics method followed by a photopolymerization process to create a crosslinked network with spatially varying composition of two hyperbranched macromers (P1000MA and H30MA) with a PEGDM. The injection of a solution of P1000MA or H30MA at 8 wt % in PEGDM and another of pure PEGDM afforded a substrate with a linear gradient of multifunctional crosslinker concentration from 0 to 8 wt %. The final sample dimensions were approximately 2 mm in width, 70 mm in length, and 1 mm thick. Distribution of the copolymer composition and final methacrylate group conversion were studied and quantified using micro-NIR. Figure 1 shows the NIR spectra of the photopolymerized PEGDM networks with a gradient in concentration of P1000MA with increasing the distance from the origin. Spectra were recorded at 0.5-mm intervals along the composition gradient. The characteristic absorption bands located at 4743 and 6162 cm^−1^ represent the methacrylate double bond stretch, and the decrease in height and area is proportional to the methacrylate conversion. From this data, the total double bond conversion was quantitatively determined by taking NIR spectra before and after curing. The methacrylate absorbance at 6162 cm^−1^ is well resolved, compared with the 4743 cm^−1^ peak that overlaps with other peaks; therefore, the 6162 cm^−1^ peak area was used for the methacrylate conversion and an internal standard reference peak was not used. The precision of the integration method was tested by comparing these conversion data with those from Mid-IR spectroscopy. Measurements in MIR monitored the decrease in intensity of the methacrylate C=C stretching mode absorption at 1637 cm^−1^, using as internal reference the area of the carbonyl peak at 1730 cm^−1^. Good correlation between the conversion data from the two IR methods was found. As shown in Figure 1, the peak size at 6162 cm^−1^ increases along the material sample, indicating that the degree of conversion is reduced with increased hyperbranched crosslinker concentration. When comparing these values with conversions obtained from uniform samples,^31^ the positions on the gradient with the same hyperbranched crosslinker concentration showed no significant differences.

**Figure 2:**
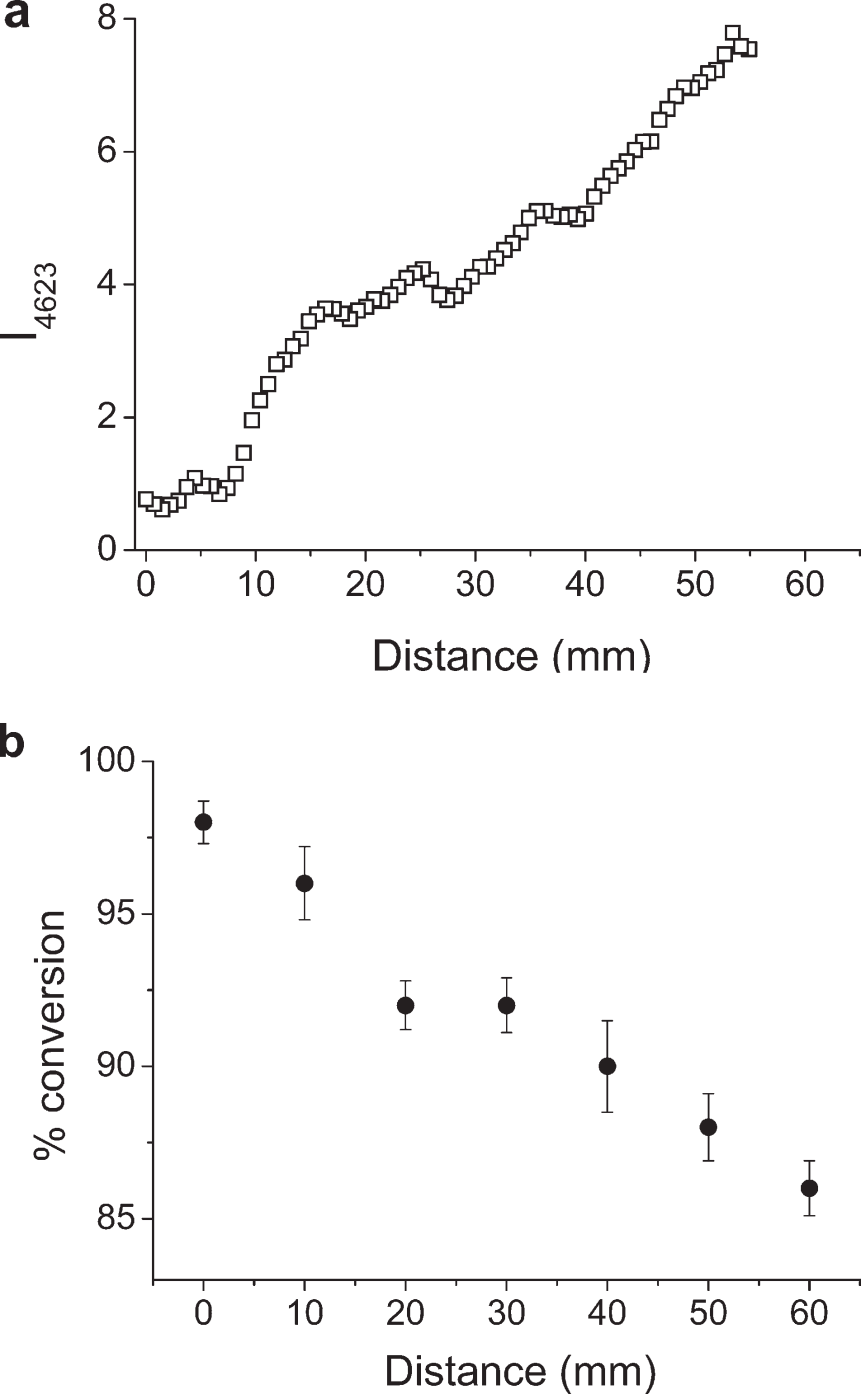
Area of the peak at 4623 cm^−1^ showing a roughly linear increase in the P1000MA content along the gradient (a). This peak corresponds to the aromatic C}H absorbance. The final methacrylate double bond conversion decreases with increasing hyperbranched crosslinker (P1000MA) content (b).

The aromatic C}H absorption band at 4623 cm^−1^ was also quantified along the sample as a measure of the composition gradient profile. The band area of the phenyl hydrogen provided an indication of the slope in the composition gradient, and demonstration of the successful achievement of the comonomer gradient [Fig. 2(a)]. The spatial distribution of the hyperbranched macromer is expected to be modulated by its diffusion and rate of reaction during the crosslinking process. With respect to the conversion of the methacrylate functional groups, the differences between the highest and the lowest conversions were statistically different, ranging from approximately 98 to 86% as shown in Figure 2(b). The increase in the unreacted double bonds as a function of P1000MA composition was accompanied by an increase in the band area at 4623 cm^−1^. Conversions for H30MA systems displayed a similar behavior with a steeper decrease after 1 wt % macromer (∼20 mm), and varied from 98 to 87%. The presence of a higher concentration of hydroxyl groups increases the viscosity leading to a more pronounced decrease in the final conversion. This decrease in the reaction extent is reflected in the final Young's modulus.

The corresponding mechanical properties (hardness and elastic modulus) were characterized using nanoindentation, a versatile technique suitable for exploring mechanical properties in localized small volumes. This technique allows measurements in the nanoscale on heterogeneous materials, and it well suited for characterizing local property variations in a single material. The methacrylate concentration, elastic modulus and hardness along the composition gradients are summarized in Tables I and II. It is apparent that the ultimate reaction conversion depends on the chemical structure of the co-monomers. There is a slight increase in the concentration of crosslinkable groups with the distance along the sample; however, the final conversion decreases. This is a consequence of a reduction in the free volume fraction, increasing the crosslink density and rigidity, and is manifested in the corresponding hardness. The presence of a hyperbranched macromer from a concentration of 3 wt % (20 mm) and higher significantly reduces the compressive moduli. These results may be explained according to the decrease in the concentration of reactive groups that soften the network structure, making the material more elastic.

**Table I:** Composition of Gradient Samples Formed From the Photopolymerization of PEGDM With the Hyperbranched Macromer P1000MA

Position (mm)	[C{C]_total_^a^ (M)	[C{C]_crossl_^b^ (M)	[C{C]_reac_^c^ (M)	*E*_r_ (MPa)	*H* (MPa)
0	4.00	2.00	3.92	114 ± 10	12 ± 1
20	3.99	2.02	3.67	65 ± 9	15 ± 1
30	3.98	2.04	3.66	85 ± 3	18 ± 1
40	3.97	2.05	3.57	90 ± 2	12 ± 0
60	3.96	2.07	3.41	100 ± 5	22 ± 1

Moduli and hardness were measured as a function of position.

a Total concentration of double bonds in the formulation.

b Concentration of crosslinkable double bonds.

c Concentration of reacted double bonds based on the conversion data.

**Table II:** Composition of Gradient Samples Formed From the Photopolymerization of PEGDM With the Hyperbranched Macromer H30MA

Position (mm)	[C{C]_total_^a^ (M)	[C{C]_crossl_^b^ (M)	[C{C]_reac_^c^ (M)	*E*_r_ (MPa)	*H* (MPa)
0	4.00	2.00	3.92	114 ± 5	12 ± 1
20	4.00	2.02	3.92	65 ± 7	7 ± 1
30	4.01	2.08	3.65	69 ± 5	8 ± 1
40	4.01	2.11	3.61	85 ± 3	9 ± 1
60	4.02	2.15	3.50	68 ± 3	3 ± 1

Moduli and hardness were measured as a function of position.

a Total concentration of double bonds in the formulation.

b Concentration of crosslinkable double bonds.

c Concentration of reacted double bonds based on the conversion data.

**Figure 3:**
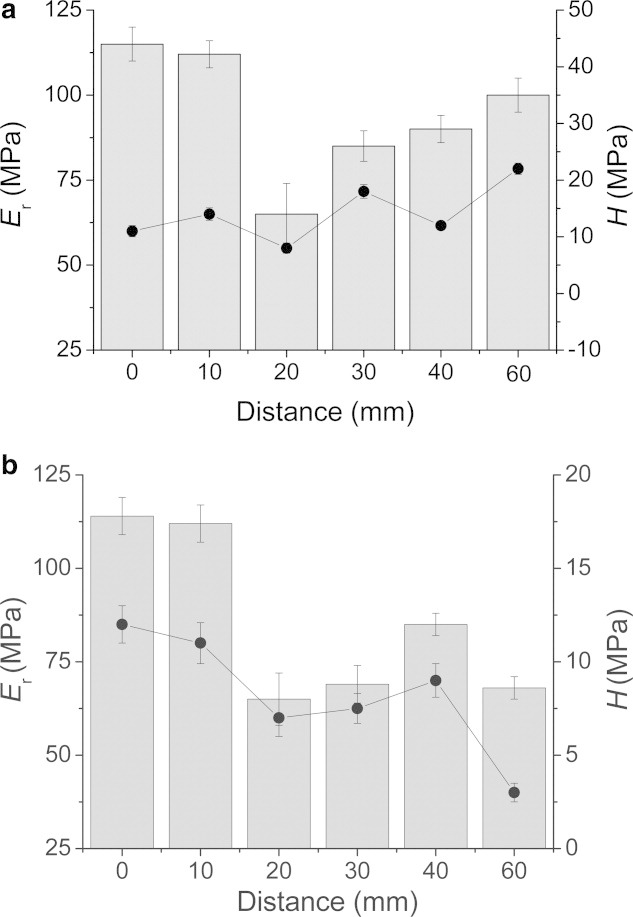
The reduced elastic modulus (bars graph) and hardness (symbol plot) as a function of position along the photopolymerized PEGDM/P1000MA (a) and PEGDM/H30MA (b) material gradients calculated from contact stiffness measurements with nanoindentation.

Figure 3 shows the differences in mechanical properties along the composition gradient. The end region with the lowest concentration of P1000MA exhibited the highest modulus [Fig. 3(a)], and the stiffness decreased sharply when the hyperbranched crosslinker content increased to about 2 wt % (10 mm). The globular end-functionalized structure of hyperbranched polymers gives rise to a lower degree of entanglement (lower viscosity) and an enhancement of reactivity. Therefore, in the PEGDM network studied, P1000MA can be considered a reactive diluent that reduces the elastic modulus. Co-monomer compositions containing higher PEGDM contents also have a higher number of methacrylates per volume. However, there is an increase of the modulus as increasing P1000MA concentration for the gradient end with the highest concentration of P1000MA; this is due to changes in the network crosslink density that increases for a higher content of the pentafunctional monomer P1000MA as a consequence of the increase in the local concentration of crosslinkable groups. Equally important is the evolution of the hardness (*H*) with conversion, also shown in Figure 3. With an increase in conversion from 90 to 98%, the elastic modulus increased from approximately 90 to 115 MPa. Over this same conversion range, the effect on the hardness of the network was less significant, as it was invariant to the macromer concentration. This mechanical behavior may also be the consequence of structural heterogeneity of the network; it has been observed by AFM^31^ that P1000MA hyperbranched macromer induces phase segregation in several formulations. Hyperbranched P1000MA domains, covalently attached to polymeric matrix, might give rise to a decrease in hardness, as observed in Figure 3(a) for low concentrations of this multifunctional crosslinker. However, the formation of aggregates at higher concentrations provokes a slight increase in the surface stiffness. Surface segregation of highly branched polymers in blends with linear polymers has been described previously.^32^, ^33^ Because of the high number of end groups per molecule of these branched polymers, they are more attracted to the surface because it is entropically favored. In this study, hyperbranched P1000MA, the stiffer component, might be likely to segregate to the surface due to entropic reasons of the different architecture. This is consistent with the measurements of increased hydrophilicity with increasing P1000MA concentration along the gradient, as set out below in the contact angle studies. On the other hand, the Young's modulus of the PEGDM substrates decreases as H30MA concentration increases [Fig. 3(b)]. The higher density of reactive groups, as the macromer concentration increases, accelerates the gel point and the final material is more elastic. Table II lists the concentration of reactive groups in different PEGDM/H30MA formulations. The marked hardness decrease for the highest concentrations of H30MA is not related to conversion. We have also observed that H30MA segregates from the PEGDM polymer matrix during the polymerization reaction in a concentration manner (Supplemental Information Fig. S1). Therefore, we propose that the H30MA domains reduce the hardness as a consequence of the covalent bonding to the polymer matrix. Phase segregation is more marked in the intermediate positions of the gradient (∼4 wt % H30MA); this is closely related to the Young's modulus values that increased at these positions to decline again when the content to macromer reached the highest value.

**Figure 4:**
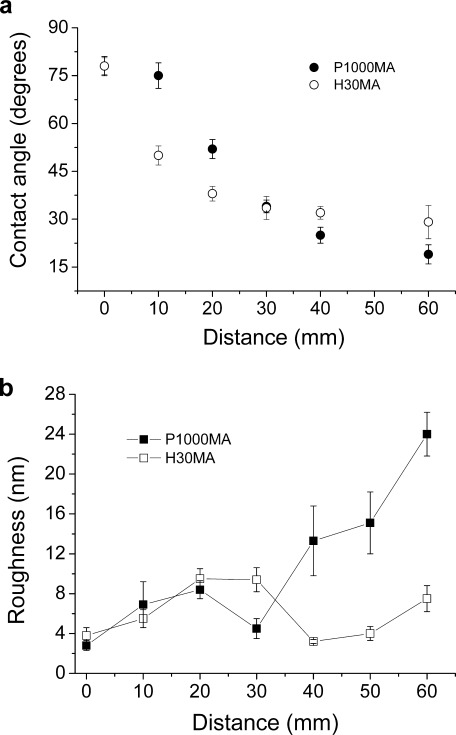
Water contact angle as a function of position along the surface composition gradients (a). Plot of the average measured roughness as a function of position for PEGDM-based materials with gradients of hyperbranched polymers P1000MA and H30MA (b), the registry is taken over domains of 20 μm.

Surface characteristics were evaluated across the gradient substrates to determine if hydrophilicity and surface roughness change as a function of composition; these are important material properties that can significantly influence cell–material interactions and ultimately cell response. For example, the adhesion and proliferation of different types of mammalian cells have been suggested to be influenced, among other things, by polymer surface wettability.^34^ Water contact angle measurements on substrates revealed significant differences as a function of conversion or crosslinker gradient. Figure 4(a) shows an increase in hydrophilicity where angle values varied from 78.0 ± 2.6° to 19.3 ± 9.5° and 29.1 ± 5.2° for low to high P1000MA and H30MA concentration, respectively. Changes in wettability are noticeable from the origin of the gradient strip in the materials with H30MA, and relate to the conversion and mechanical properties, previously discussed. Phase segregation is the cause of the incomplete conversion and depleted mechanical properties. The largest differences observed between the two macromers are in the first half of the gradient, where the concentrations of the multifunctional crosslinker are lower and where differences in phase segregation are more marked (Fig. S1).

Further, the topography as a function of substrate position was measured using AFM, and results are plotted in Figure 4(b). The increase in P1000MA concentration was related to an increase in the rms roughness, finding the sharpest increase toward the end of the gradient. AFM measurements of rms roughness ranged from 2.5 ± 0.4 nm at 98% conversion to 24.0 ± 2.1 nm at 85% conversion. With contact angles lower than 90°, a decrease with increasing roughness was explained by a capillarity effect.^35^ Figure 4(b) presents the variation in roughness along the gradient when PEGDM was copolymerized with the different hyperbranched macromers. It is worthy to point out that H30MA is a hyperbranched polyester that has an average of 16 methacrylate groups per branched macromolecule. The smaller changes in roughness along the gradient, when compared with P1000MA data, are likely related to aggregate formation. This effect has been observed by AFM in PEGDM based materials in the presence of P1000MA (Fig. S1). The π–π* stacking interactions between phenyl groups of P1000MA can drive the self-assembly process to form clusters inside the acrylic matrix that migrate to surface. Formation of rigid fractions, such as P1000MA phenyl groups, can give rise to hollow cavities in between neighboring branches, in contrast to aliphatic chains of the building blocks.^36^ These results show the fabrication conditions and microstructure/topography ranges that should prove useful in future studies aimed to study cellular response to these heterogeneous substrate surfaces.

**Figure 5:**
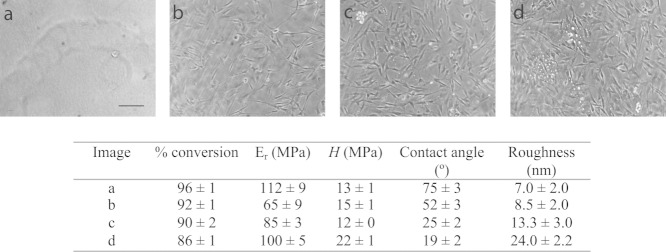
Representative light micrographs of valvular interstitial cells (VICs) attached (48 h after seeding) to the surface of photopolymerized samples fabricated from PEGDM with a gradient concentration of hyperbranched multimethacrylate P1000MA corresponding to spatial positions of 10 (a), 20 (b), 40 (c), and 60 mm (d) along the gradient. The table summarizes the properties of the substrates as a function of the position corresponding to the location of the presented cell images. Scale bar = 50 μm.

Here, we demonstrate the usefulness of this one-dimensional gradient library of a polymer network to screen rapidly cell attachment and morphology as a function of property gradients along a single substrate. This approach can prove useful as cells on gradient materials experience essentially identical culture conditions, and the average response to the underlying substrate is measured in a more exact and precise manner, reducing the common experimental errors. Specifically, VICs were cultured on a PEGDM/P1000MA gradient material for 2 days. As observed in the light micrographs of Figure 5, VIC attachment was dependent on the hyperbranched macromer concentration with increased attachment at higher P1000MA content. Further, significant morphological changes were observed as a function of the underlying gradient material properties. Cell adhesion and proliferation were similar on both the P1000MA and H30MA rich sides of the samples. However, after incorporating 1–2 wt % of H30MA, cell adhesion is almost equal along the sample, without a gradient increase, as observed for P1000MA. This observation is also related to the abrupt change in the conversion and subsequent surface stiffness (Supplemental Information Fig. S2).

Our results revealed that there was no significant change in total protein adsorption for all concentrations of P1000MA and H30MA used (Supplemental Information Fig. S3), suggesting that cells directly respond to changes in substrate topology or rigidity.^37^ However, it has to be also considered the possibility of differential levels of protein adsorption profiles. The use of gradient substrates for cell culture also suggested a threshold value for macromer concentration on PEGDM based materials [about 6 wt %, Fig. 5(c)], about which there is a significant increase in cell attachment and spreading. This value corresponds to a modulus of 90 MPa and a water contact angle of 30°. Alternatively, phase separation can be a significant cause of increasing cell adhesion with topography, because it has been shown that substrates with roughness values of 13 nm displayed greater spreading and higher degrees of F-actin organization, compared with a control substrate with 1 nm roughness value.^38^ Other effects that may relate to roughness and cell adhesion are the conformation of adherent serum proteins, which have similar sizes to the topography. However, because we have no evidence of differences in irreversible protein adsorption along our gradient substrate (Fig. S3), the driving force of cell spreading must fall on other surface characteristics like mechanics or hydrophobicity. Although cell adhesion is normally higher in hydrophobic surfaces, a balance between hydrophobicity and hydrophilicity is normally necessary for good cell spreading; explaining the lack of cell adhesion on the PEG enriched side (a). The decline in conversion of vinyl groups enhances the chain mobility on the surface, which can act as a promoter of cell adhesion,^39^ as well as being responsible of the slight increase in the swelling ratio with the concentration of P1000MA. Although all these factors described above have an influence on cell–material interactions, surface hardness and elasticity are of equal or greater importance, as it cannot be overlooked that cells have shown the preference to migrate toward stiffer regions of the substrate.

## CONCLUSIONS

A simple microfluidics methodology followed by a photopolymerization reaction was used to generate materials with controlled gradient network properties on the microscale. The presence of hyperbranched macromers was used to tune the monomer (and final polymer) composition, but also altered the methacrylate conversion (85–96%). Double bond conversion was measured using near IR spectroscopy, and the mechanical properties (i.e., elastic modulus and hardness) were determined using nanoindentation with a continuous stiffness method. Relationships between the chemical composition and surface properties (roughness and hydrophilicity) were also evaluated. With this technique, we were able to evaluate the impact of monomer composition on final conversion, taking into account the reactivity, functionality and viscosity changes during the photopolymerization reaction. The addition of the P1000MA diluent macromonomer increased roughness while decreasing the water contact angle. The high concentration of reactive groups in H30MA reduces the final conversion and makes the materials more compliant. The present technique allows several properties to be varied and characterized on a single substrate platform. Data obtained using this type of high-throughput approach is useful in providing information related to structure–properties relationships as a function of composition, and ultimately cell–material interactions. Such knowledge should prove useful toward the development of new material systems and formulations with tunable properties on the microscale for controlling cell function.

## Notes

Supporting Figure 1 AFM phase images of the surfaces of gradients of PEGDM with H30MA (a) and P1000MA (b). Images are taken at 10, 30 and 60 mm from the origin in the sample strips. Phase segregation is evident in both (a) and (b), however, P1000MA appears as the hardest component while H30MA does as the softest and present a higher heterogeneity in the middle point of the sample.

Supporting Figure 2 Light micrographs of VICs attached (48h after seeding) to the surface of PEGDM / H30MA gradient materials. Images correspond to the special positions of 10 (a), 30 (b) and 60 (c). The presence of H30MA influences cell attachment although the increase is not concentration dependent.
