# Supplementary figures and images for: Microfluidic approaches for the fabrication of gradient crosslinked networks based on poly(ethylene glycol) and hyperbranched polymers for manipulation of cell interactions
Source: J Biomed Mater Res A. 2011 Jan;96A(1):196–203. doi: 10.1002/jbm.a.32974 (PMC3059081; doi:10.1002/jbm.a.32974)

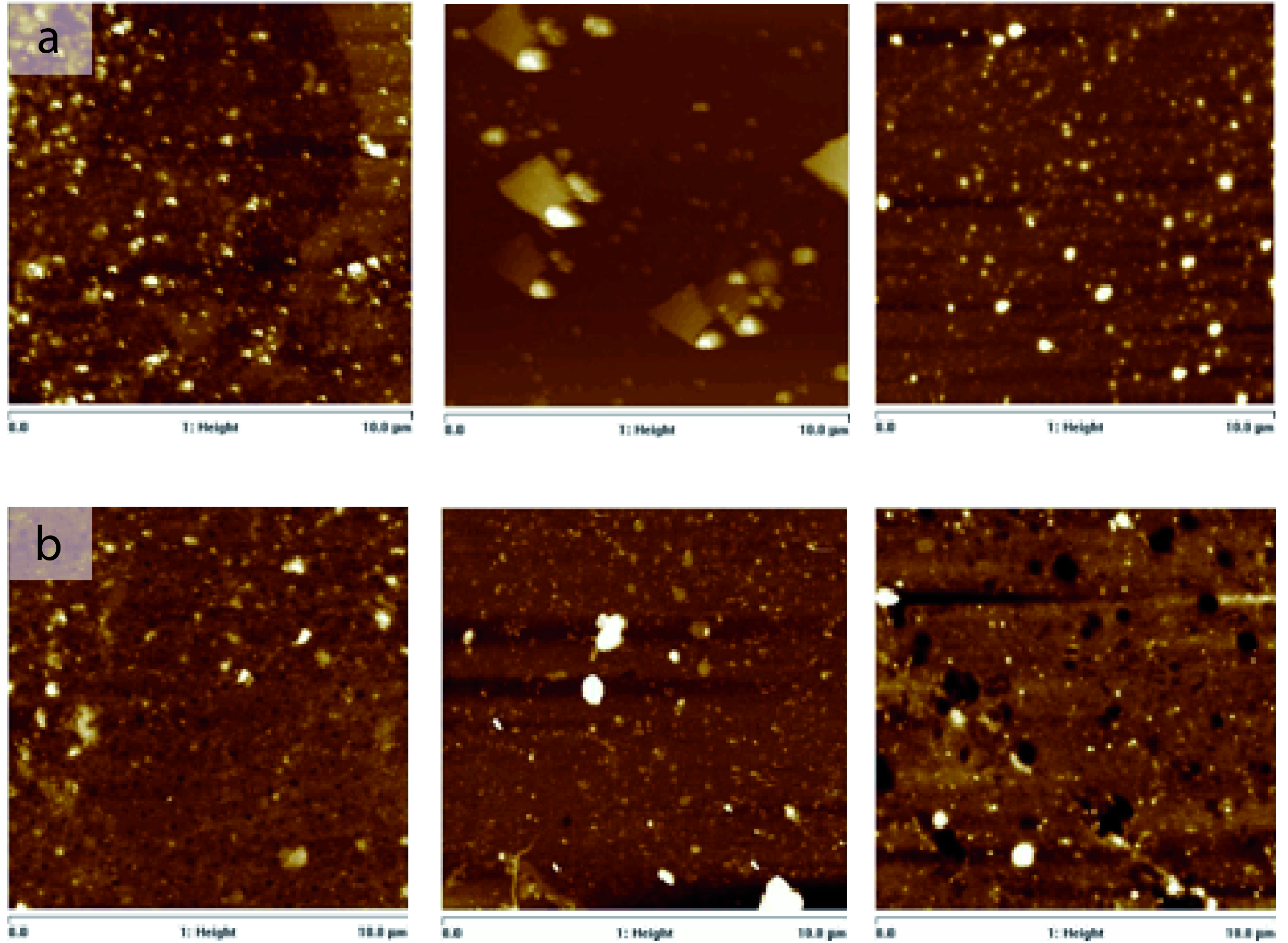

Supplement: Supporting Figure 1 — AFM phase images of the surfaces of gradients of PEGDM with H30MA (a) and P1000MA (b). Images are taken at 10, 30 and 60 mm from the origin in the sample strips. Phase segregation is evident in both (a) and (b), however, P1000MA appears as the hardest component while H30MA does as the softest and present a higher heterogeneity in the middle point of the sample. [file jbm0096-0196-sd1.tif]

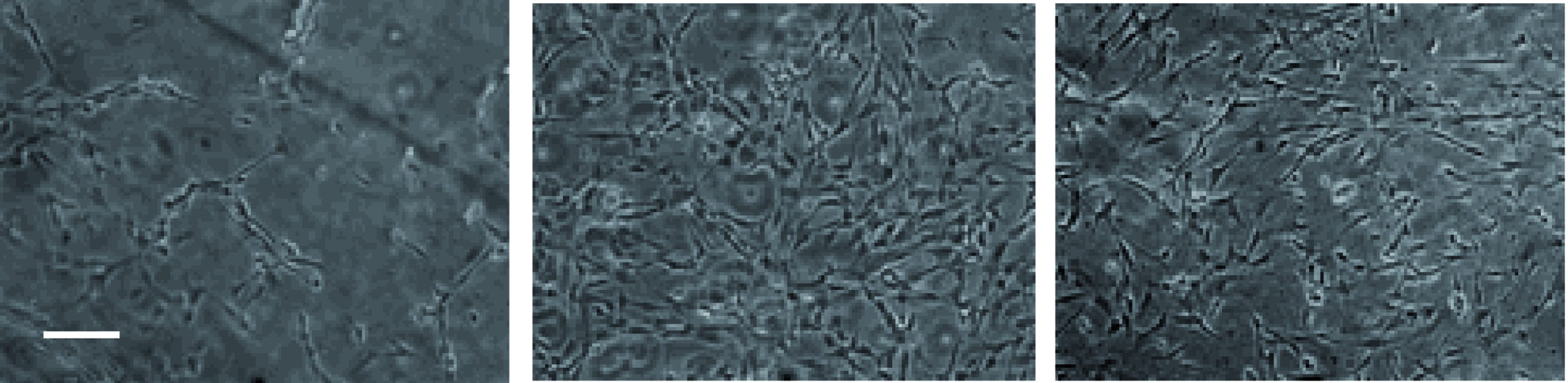

Supplement: Supporting Figure 2 — Light micrographs of VICs attached (48h after seeding) to the surface of PEGDM / H30MA gradient materials. Images correspond to the special positions of 10 (a), 30 (b) and 60 (c). The presence of H30MA influences cell attachment although the increase is not concentration dependent. [file jbm0096-0196-sd2.tif]

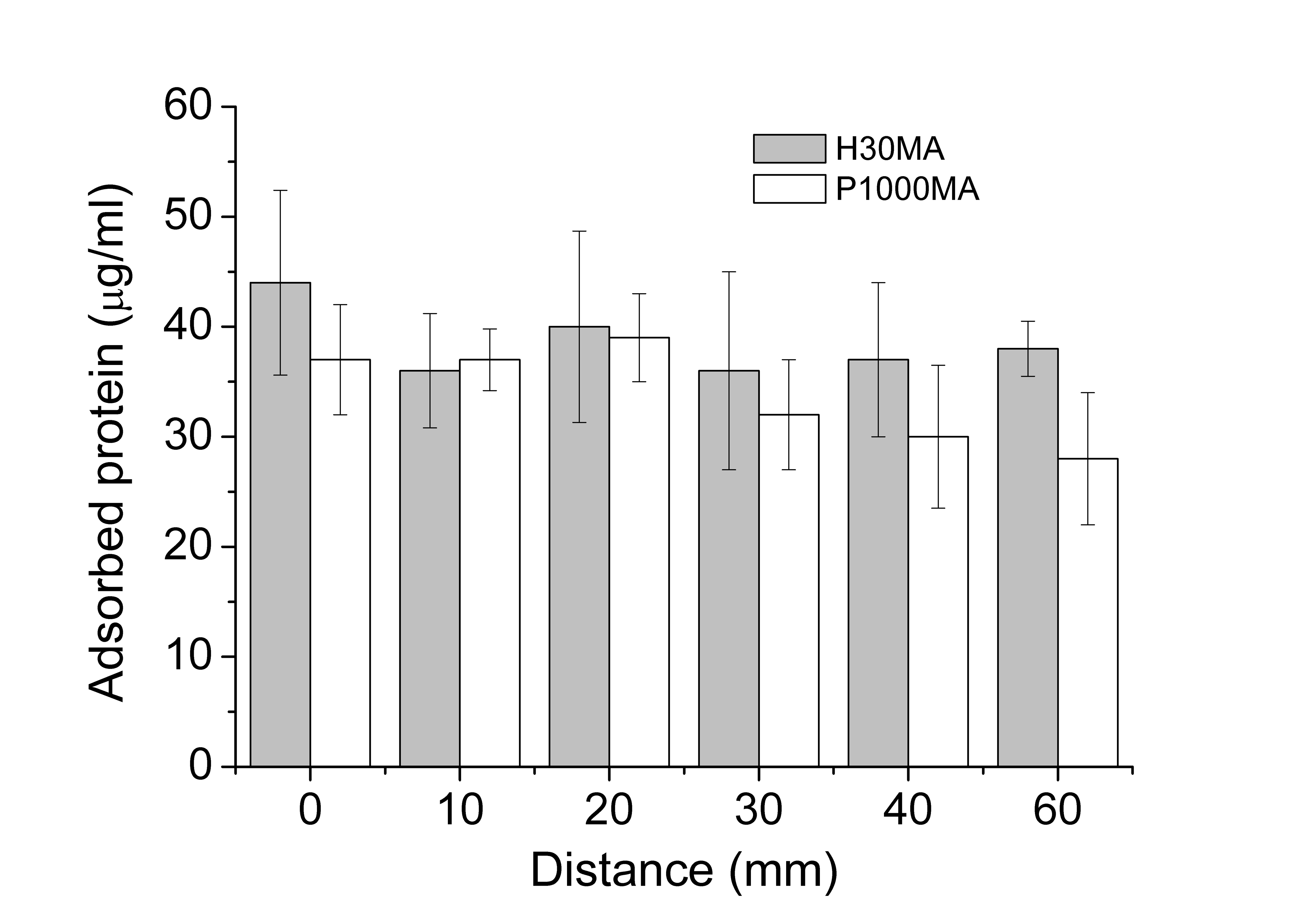

Supplement: Supplementary file 3 [file jbm0096-0196-sd3.tif]
